# The Effects of Sodium-Glucose Cotransporter-2 Inhibitors (SLGT-2i) on Cardiovascular and Renal Outcomes in Non-diabetic Patients: A Systematic Review

**DOI:** 10.7759/cureus.25476

**Published:** 2022-05-30

**Authors:** Suganya Giri Ravindran, Meghana Kakarla, Musa Ausaja Gambo, Mustafa Yousri Salama, Nathalie Haidar Ismail, Pardis Tavalla, Pulkita Uppal, Shaza A Mohammed, Shriya Rajashekar, Pousette Hamid

**Affiliations:** 1 Internal Medicine, California Institute of Behavioral Neurosciences & Psychology, Fairfield, USA; 2 Research, California Institute of Behavioral Neurosciences & Psychology, Fairfield, USA; 3 Neurology, California Institute of Behavioral Neurosciences & Psychology, Fairfield, USA

**Keywords:** dapagliflozin, empagliflozin, sglt-2 inhibitor, non-diabetes, diabetes, renal effects, cardiovascular effects, sodium-glucose cotransporter-2 (sglt-2) inhibitors

## Abstract

Globally, cardiovascular disease (CVD) and chronic kidney disease (CKD) are the leading causes of mortality. Despite medical advances, these illnesses are still underdiagnosed and undermanaged. Sodium-glucose cotransporter-2 inhibitors (SGLT-2i) have recently emerged as a potential class of medications with promising cardiovascular and renal safety in non-diabetic patients. In this systematic review, we explored the outcomes of cardiovascular and renal protective effects utilizing SGLT-2i in three large randomized clinical trials with a cohort of both diabetes and non-diabetes patients. In these studies, data conferred that there is a significant reduction in heart failure (HF) hospitalization, as well as cardiovascular and all-cause mortality. Moreover, SGLT-2i impede the progression to and death from CKD. Additionally, we reviewed trials solely done on non-diabetics which demonstrated benefits in patients with established HF with reduced ejection fraction, though the fact that these studies had a smaller sample size. We also discussed some of the potential mechanisms of action of SGLT-2i on cardiovascular and renal outcomes that are beyond anti-hyperglycemic control. There is ongoing research involving a larger number of non-diabetes patients that may provide more information about the efficacy of these drugs besides anti-diabetic medications in the future. Finally, this is the first systematic review that has provided a perspective on the currently available trials, which offer evidence supporting the potential benefits of SGLT-2i on cardiovascular and renal outcomes in non-diabetic individuals.

## Introduction and background

According to the Global Burden of Disease (GBD), cardiovascular disease (CVD) and chronic kidney disease (CKD) are the leading causes of global mortality [1]. Despite breakthroughs in medicine, heart failure (HF) is a primary cause of hospitalization, mortality, and morbidity, as well as low quality of life [2]. Moreover, HF patients are frequently associated with comorbidities, such as CVD and CKD [3]. CKD affects an estimated 840 million individuals globally [4]. As the condition progresses, it can lead to adverse clinical outcomes, and eventually, kidney failure [5]. Unfortunately, the standard therapy for HF has been unaltered for years which includes diuretics, beta-blockers, and renin-angiotensin system inhibitors. Even though these drugs offer cardioprotective and renoprotective benefits, mortality rates continue to grow [6]. Therefore, it is paramount to emphasize on medications that can assist to mitigate these adverse outcomes.

Sodium-glucose cotransporter-2 inhibitors (SGLT-2i) were first discovered as a novel anti-diabetic drug [7]. Besides glucose-lowering actions, recent trials have indicated that SGLT-2i have demonstrated its efficacy in reducing the adverse cardiovascular (CV) and renal outcomes in diabetic patients [8-11]. Interestingly, it has garnered attention in recent years for its extended benefits beyond the anti-hyperglycemic actions, such as lowering the risk of hospitalization for HF, cardiovascular and all-cause mortality, and death from CKD, regardless of the baseline diabetic status. With a few mechanisms of action, SGLT-2i effects have proven to be substantially independent of or nonetheless separated from the effects of blood glucose values [12,13]. The pathophysiology through which SGLT-2i exert combined cardiorenal protective outcomes is still unclear. In regard to the cardiac effects, the postulated mechanisms of action are activation of anti-inflammatory and oxidative stress pathways, increased ketone body protection, and reduction in advanced glycation end products [2,14].

As a result of growing evidence of SGLT-2i’s prognostic benefits, further research is ongoing to uncover its benefits on CV and renal outcomes in large cohorts, extending to non-diabetic patients. Initially, two large randomized trials, the Dapagliflozin and Prevention of Adverse Outcomes in Heart Failure (DAPA-HF) and the Empagliflozin Outcome Trial in Patients with Chronic Heart Failure with Reduced Ejection Fraction (EMPEROR-REDUCED), showed a significant reduction in incidents of heart failure events and major renal outcomes with a cohort consisting of both diabetics and non-diabetics [15-17]. Another trial, the Dapagliflozin and Prevention of Adverse outcomes in Chronic Kidney Disease (DAPA-CKD), contributed renoprotective effects by a sustained decline in estimated glomerular filtration rate (eGFR), end-stage renal disease (ESRD), and renal death, irrespective of the diabetic status [18].

In response to the aforementioned trials, an increasing number of studies are reporting on SGLT-2i in non-diabetic patients. The purpose of this systematic review is to combine all available evidence on the combined cardioprotective and renoprotective outcomes of SGLT-2i in a substantial number of non-diabetic individuals in order to better understand the therapeutic rationale for their usage in those cohorts. Additionally, we have included studies with both cohorts, diabetes and non-diabetes, and also trials exclusively done on diabetic individuals, to evaluate the efficacy and to raise future research questions in this area.

## Review

Methodology

We conducted our systematic review using Preferred Reporting Items for Systematic Reviews and Meta-analyses (PRISMA) guidelines [19]. We adhered to the PRISMA 2020 recommendations [20,21].

Database

We systematically started our articles search in December 2021, using two online databases, PubMed and Google Scholar, for data collection.

Search Strategy

We incorporated the building block technique for search strategy and explored studies related to sodium-glucose cotransporter-2 inhibitors (SGLT-2i)/SGLT-2 inhibitors on cardiovascular outcomes/SGLT-2 inhibitors on renal outcomes/diabetes. Our keywords and medical subject heading (MeSH) words included SGLT-2 inhibitors, cardiovascular outcomes, renal outcomes, heart failure, chronic kidney disease, and diabetes. We have included the regular keywords and MeSH keywords in the search (Table 1).

**Table 1:** Keywords utilized in the study search. MeSH: medical subject headings; SGLT-2i: sodium-glucose cotransporter-2 inhibitors.

Search strategy	Keywords
Regular keywords	Sodium-Glucose Cotransporter-2 Inhibitors; cardiovascular outcomes; renal outcomes; empagliflozin; Dapagliflozin; diabetes; heart failure; chronic kidney disease
MeSH keywords	Dapagliflozin OR anti-hyperglycemics OR sodium-glucose cotransporter 2 OR lower blood sugar OR empagliflozin OR canagliflozin AND Heart failure OR chronic heart disease OR acute heart failure OR chronic kidney disease OR acute kidney disease OR eGFR OR creatinine OR renal failure OR cardiovascular death OR non fatal myocardial infarction OR hospitalization OR kidney function OR atherosclerosis AND (( "Sodium-Glucose Transporter 2 Inhibitors/administration and dosage"[Majr] OR "Sodium-Glucose Transporter 2 Inhibitors/pharmacology"[Majr] OR "Sodium-Glucose Transporter 2 Inhibitors/physiology"[Majr] OR "Sodium-Glucose Transporter 2 Inhibitors/therapeutic use"[Majr] OR "Sodium-Glucose Transporter 2 Inhibitors/toxicity"[Majr] )) OR ( "Sodium-Glucose Transporter 2 Inhibitors/administration and dosage"[Mesh:NoExp] OR "Sodium-Glucose Transporter 2 Inhibitors/pharmacology"[Mesh:NoExp] OR "Sodium-Glucose Transporter 2 Inhibitors/physiology"[Mesh:NoExp] OR "Sodium-Glucose Transporter 2 Inhibitors/therapeutic use"[Mesh:NoExp] OR "Sodium-Glucose Transporter 2 Inhibitors/toxicity"[Mesh:NoExp] ) AND (( "Cardiovascular System/complications"[Majr] OR "Cardiovascular System/drug effects"[Majr] OR "Cardiovascular System/therapeutic use"[Majr] OR "Cardiovascular System/toxicity"[Majr] )) OR ( "Cardiovascular System/complications"[Mesh:NoExp] OR "Cardiovascular System/drug effects"[Mesh:NoExp] OR "Cardiovascular System/therapeutic use"[Mesh:NoExp] OR "Cardiovascular System/toxicity"[Mesh:NoExp] ) AND (( "Kidney Diseases/complications"[Majr] OR "Kidney Diseases/drug effects"[Majr] OR "Kidney Diseases/drug therapy"[Majr] OR "Kidney Diseases/metabolism"[Majr] OR "Kidney Diseases/pharmacology"[Majr] OR "Kidney Diseases/physiology"[Majr] OR "Kidney Diseases/therapeutic use"[Majr] )) OR ( "Kidney Diseases/complications"[Mesh:NoExp] OR "Kidney Diseases/drug effects"[Mesh:NoExp] OR "Kidney Diseases/drug therapy"[Mesh:NoExp] OR "Kidney Diseases/metabolism"[Mesh:NoExp] OR "Kidney Diseases/pharmacology"[Mesh:NoExp] OR "Kidney Diseases/physiology"[Mesh:NoExp] OR "Kidney Diseases/therapeutic use"[Mesh:NoExp] )

Inclusion Criteria

We looked at peer-reviewed articles and studies from the last 10 years that were published in English. Only human research in the categories of systematic reviews, meta-analyses, and randomized clinical trials focusing on adults >18 years and geriatrics were considered. The eligible papers were screened using the Population, Intervention, Comparison, and Outcomes (PICO) model. 

Exclusion Criteria

Gray literature, unpublished articles, case reports, and animal research were omitted. We also did not include studies written prior to 2011.

Data Extraction

Two researchers worked independently on data retrieval and extraction. If a decision cannot be made due to a conflict of interest, a third reviewer is approached.

Quality Assessment Tools

We used the Cochrane risk bias assessment tools for randomized clinical trials to critically evaluate our papers [22]. The assessment of multiple systematic reviews (AMSTAR) checklist was reviewed for systemic review and meta-analysis [23]. We omitted research that was of poor quality or unrelated to the subject.

Results

A total of 10,880 records were found using regular keywords and MeSH keywords, databases, and eligibility criteria. From pertinent journals, 10,000 were found in PubMed, and 880 were found in Google Scholar. After EndNote (Clarivate 1.9, Philadelphia, United States) was used to remove duplicates (n=34), 5,392 unique articles were searched by titles and abstracts. A further 3,452 articles were eliminated owing to their topic that is irrelevant. Of the remaining articles, a total of 1,940 articles were sought for retrieval. Following that, 314 papers were assessed for eligibility criteria, and an additional 305 articles were removed due to the risk of bias, low quality, and other factors. The final criteria were met by nine articles, including seven randomized controlled trials (RCTs)/observational studies, one systematic review, and one systematic review and meta-analysis. The characteristics of the individual studies with analysis and outcome measures included in this systematic review are presented in Table 2.

**Table 2:** A summary of final studies in data extraction. DAPA: dapagliflozin; DAPA-HF: Dapagliflozin and Prevention of Adverse Outcomes in Heart Failure; DAPA-CKD: Dapagliflozin and Prevention of Adverse Outcomes in Chronic Kidney Disease; DEFINE-HF: Dapagliflozin Effects on Biomarkers, Symptoms, and Functional Status in Patients With HF with Reduced Ejection Fraction; EMPA: empagliflozin; EMPEROR-REDUCED: Empagliflozin Outcome Trial in Patients With Chronic Heart Failure With Reduced Ejection Fraction; EMPIRE-HF: Empagliflozin in Heart Failure Patients with Reduced Ejection Fraction; EMPA-TROPISM: Empagliflozin in Non-diabetic Patients With Heart Failure and Reduced Ejection Fraction; DIAMOND: Effects of the SGLT2 Inhibitor Dapagliflozin on Proteinuria in Non-diabetic Patients With Chronic Kidney Disease; CANA: canagliflozin; SOTA: sotagliflozin; ERTU: ertugliflozin; RCT: randomized clinical trial; HF: heart failure; EF: ejection fraction; CKD: chronic kidney disease; NT-proBNP: non-terminal prohormone B-type natriuretic peptide; HFrEF: heart failure with reduced ejection fraction; LV: left ventricular; QoL: quality of life; SGLT-2i: sodium-glucose cotransporter-2 inhibitors; CV: cardiovascular; eGFR: estimated glomerular filtration rate; AMSTAR: assessment of multiple systematic reviews.

Author	Year	Quality appraisal tool used	SGLT-2 inhibitor	Study design	Total sample size	Sample size of non-diabetics	Aim	Conclusion
Petrie et al. [16]	2020	Cochrane risk of bias assessment tool	DAPA	RCT (DAPA-HF)	4,744	2,605	To assess if DAPA affects cardiovascular outcomes in patients with HF and a low EF, regardless of whether or not they are diabetic.	Reduction in worsening of HF and CV death was noted in non-diabetics similar to diabetics in addition to the recommended therapy.
Packer et al. [17]	2020	Cochrane risk of bias assessment tool	EMPA	RCT (EMPEROR-REDUCED)	3,730	606, pre-diabetes 1,268	To get evidence of EMPA in a wide range of HF patients with significantly reduced EF.	EMPA lowered the risk of CV death and the hospitalization for HF regardless of the presence or absence of diabetes.
Heerspink et al. [24]	2016	Cochrane risk of bias assessment tool	DAPA	RCT (DAPA-CKD)	4,304	32.50%	To identify the efficacy of DAPA in CKD in both diabetes and non-diabetics.	DAPA showed a sustained decline in the eGFR of at least 50%, end-stage kidney disease, and death from renal or CV causes in both diabetes and non-diabetes individuals.
Nassif et al. [18]	2019	Cochrane risk of bias assessment tool	DAPA	RCT (DEFINE-HF)	236	70	To establish the effect of DAPA in established HF with reduced EF in patients with or without diabetes.	The advantages of DAPA on clinically meaningful HF measures in patients with HFrEF extend to patients without diabetes as well.
Cherney et al. [25]	2020	Cochrane risk of bias assessment tool	DAPA	RCT (DIAMOND TRIAL)	53	53	To assess the efficacy of DAPA in patients with proteinuric kidney disease without diabetes.	No impact on proteinuria in patients with kidney disease.
Jensen et al. [26]	2020	Cochrane risk of bias assessment tool	DAPA	RCT (EMPIRE-HF)	190	Both diabetes and non-diabetes	To investigate the effects of EMPA in NT-proBNP in patients with HFrEF.	EMPA did not change NT-proBNP after 12 weeks regardless of diabetic status.
Santos-Gallego et al. [27]	2021	Cochrane risk of bias assessment tool	EMPA	RCT (EMPA-TROPISM)	84	84	To identify the benefits of EMPA on LV function and volume, functional capacity, and QoL in non-diabetic HFrEF.	Considerable improvement in LV volume, LV mass, LV systolic function, functional capacity, and QoL.
Zannad et al. [28]	2020	AMSTAR 2 checklist	DAPA and EMPA	Meta-analysis	8,474	4,479	To assess the effects of SGLT-2i in patients with HFrEF with or without diabetes.	Evidence on reduction in hospitalization for HF and suggested to reduce all-cause and CV mortality and improve renal outcomes regardless of diabetic status.
Tsampasian et al. [29]	2021	AMSTAR 2 checklist	DAPA, EMPA, CANA, SOTA, ERTU	Systematic review and meta-analysis	13,275	4,576	To gather evidence of SGLT-2i effectiveness in patients with HF irrespective of their baseline diabetes status.	Considerable prognostic benefit observed in patients with HF which extends to non-diabetic subjects as well.

**Figure 1:**
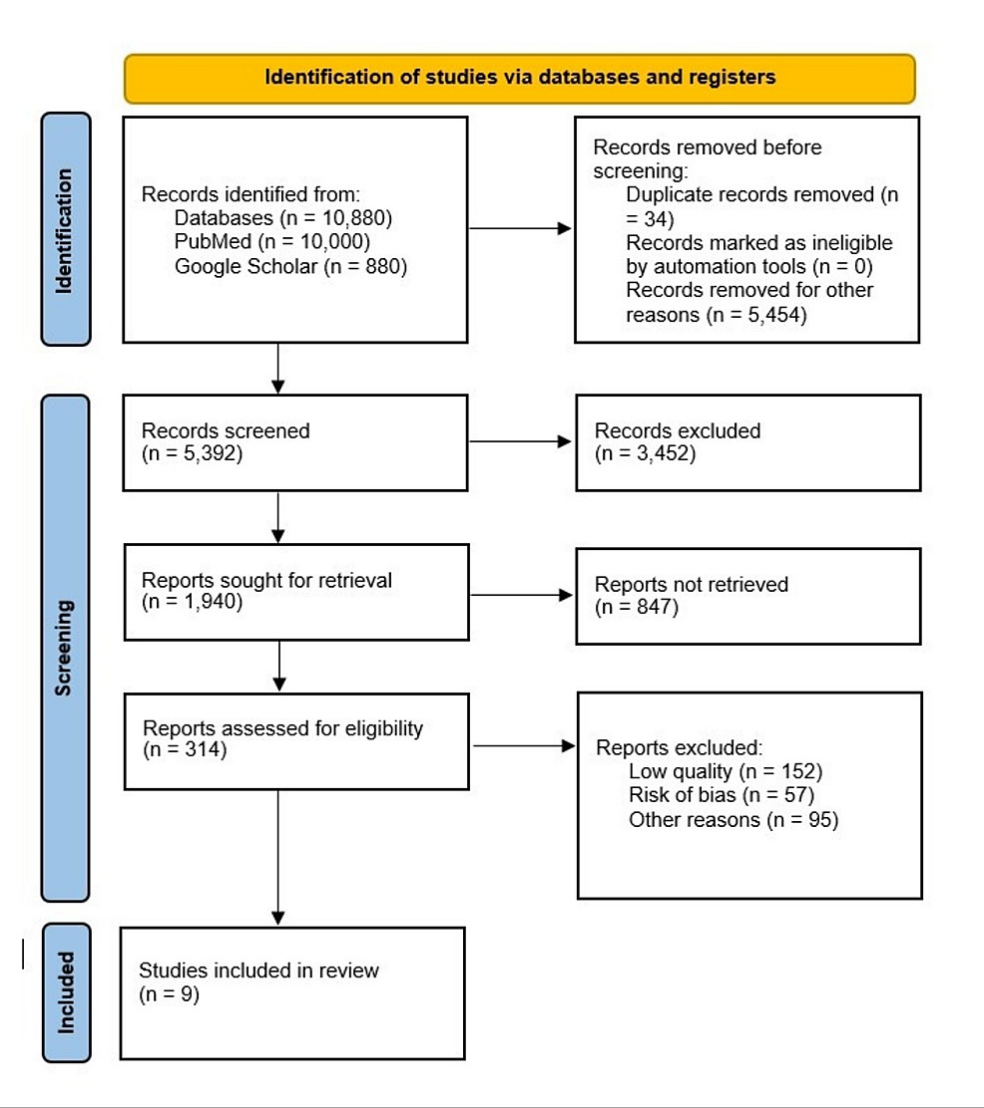
Comprehensive PRISMA flow diagram. PRISMA: Preferred Reporting Items for Systematic Reviews and Meta-analyses.

We have presented our PRISMA flow diagram in Figure 1.

Discussion

In this review, we will cover the likely mechanisms of action and current evidence available in the non-diabetic patient cohorts. We will also analyze differences in various trials in terms of cardiovascular and renal outcomes, limitations, and future recommendations, to throw light on future research questions.

Mechanisms of Action of Sodium-Glucose Cotransporter-2 Inhibitors (SGLT-2i) in Cardiorenal Protective Outcomes

While the specific mechanism by which SGLT-2i exert its cardioprotective and renoprotective effects is not fully understood, numerous hypotheses have been proposed to provide evidence that SGLT-2i have functions other than glycemic regulation. Some of the postulated mechanisms of action are discussed here. The benefits of SGLT-2i in heart failure (HF) are mostly attributable to the stimulation of natriuresis and osmotic diuresis that these agents stimulate [12,30]. Increased ketogenesis leads to a rise in hematocrit, which increases oxygen delivery to the vascular bed, such as the renal medulla and myocardium [31]. Moreover, SGLT-2i have been shown to reduce myocardial workload by lowering blood pressure, and there are data to suggest that reduction in arterial stiffness and improvement in vascular resistance might also play a significant role [13,32]. Another positive characteristic is the antifibrotic effect on the heart; empagliflozin (EMPA) has demonstrated its efficacy in abating the myocardia fibrosis after myocardial infarction (MI) and attenuating the myofibroblast activity and cardiac remodeling [33,34]. In addition, SGLT-2i regulate the tubuloglomerular feedback and reduce the intraglomerular pressure and hyperfiltration by releasing adenosine in the proximal convoluted tubules [24].

Current Evidence on Cardiorenal Benefits of SGLT-2i, Limitations, and Recommendations

The first large breakthrough randomized clinical trial (RCT) of SGLT-2i that included both diabetes and non-diabetes is the Dapagliflozin and Prevention of Adverse Outcomes in Heart failure (DAPA-HF) trial [15]. DAPA-HF enrolled 4,744 patients, 2,605 of whom did not have diabetes. Those enrolled in this trial had HF and a low ejection fraction (EF) of the estimated glomerular filtration rate (eGFR) of ≥30 mL/min/1.73 m^2^. They were tracked for a median of 18.2 months, and the hazard ratio (HR) for primary outcomes of both diabetes and without diabetes was similar (HR of primary outcome, 0.75 and 0.73, respectively). This study demonstrated that dapagliflozin (DAPA) was equally effective in 55% of patients without type 2 diabetes as it was on those who did have diabetes. This was supported by the findings of an exploratory analysis of this trial in 2020 by Petrie et al., which showed a considerable reduction in the primary composite outcomes of worsening HF or cardiovascular death in patients without diabetes (HR, 0.73 in non-diabetics, 0.74 in individuals with glycated hemoglobin of at least 5.7%, and 0.67 in those with glycated hemoglobin <5.7%) [16].

Simultaneously, another major study, the Empagliflozin Outcome Trial in Patients With Chronic Heart Failure with Reduced Ejection Fraction (EMPEROR-REDUCED), replicated the findings of DAPA-HF but with a reduced eGFR threshold of 20 mL/min/1.73 m^2^ [17]. The effect of empagliflozin (EMPA) on the primary outcomes was consistent across all predefined subgroups, including diabetes and non-diabetic patients (HR, 0.72 and 0.78, respectively).

The findings of the two trials were nearly identical in both diabetic and non-diabetic individuals, such as reduced hospitalization for HF, all-cause mortality, and progression from chronic kidney disease (CKD). Furthermore, DAPA-HF and EMPEROR-REDUCED are the only trials that evaluated the effect of SGLT-2i on mortality and morbidity in patients with symptomatic heart failure reduced ejection fraction (HFrEF), increased natriuretic peptides, and patients with or without diabetes. It is worth noting that the relative reduction in cardiovascular death was lower in EMPEROR-REDUCED (8%) than in DAPA-HF (18%), which merits more investigation in the future. In terms of renal benefit, the reduction in eGFR was less in the empagliflozin group compared to the placebo group (-0.55 mL/minute/1.73 m^2^/year versus -2.28 ± 0.23 mL/min/1.73 m^2^/year; P < 0.001). To corroborate this, a prespecified analysis of the EMPEROR-REDUCED study was undertaken, which highlighted the fact that EMPA decreases heart failure and renal adverse effects in pre-diabetic and normoglycemic patients [35]. Furthermore, a meta-analysis on EMPEROR-REDUCED and DAPA-HF [28] validated the cardioprotective benefits of SGLT-2i. Additionally, the meta-analysis stated that there were no significant adverse effects of SGLT-2i and that these medications were well tolerated in both investigations.

Following that, Heerspink et al. demonstrated dapagliflozin efficacy in non-diabetics with chronic kidney disease in the Dapagliflozin on Renal Outcomes and Cardiovascular Mortality in Patients With Chronic Kidney Disease (DAPA-CKD) trial, which enrolled 4,304 patients, 32.5% of whom were non-diabetics [36]. The study was halted early due to its tremendous effectiveness. It stated unequivocally that individuals with CKD who were using dapagliflozin had a lower risk of at least 50% in diminishing in eGFR, end-stage kidney disease, or mortality from renal or cardiovascular reasons, regardless of having diabetes or not. Also, the primary outcome was similar in both diabetes and non-diabetes subjects. Especially, the effectiveness of DAPA was at best in non-diabetic kidney disease patients. Moreover, dapagliflozin has been proven to reduce worsening renal function or death from kidney failure, hospitalization for HF or cardiovascular (CV) death, and all-cause mortality. Dapagliflozin was similarly found to be safe in patients with CKD, with no changes in adverse effects when compared to placebo.

A limited number of studies examined the effects of SGLT-2i on a mixed sample of diabetics and non-diabetics. Nassif et al. enrolled 236 adult ambulatory patients with or without type 2 diabetic mellitus (T2DM) in the Dapagliflozin Effects on Biomarkers, Symptoms, and Functional Status in Patients With HF With Reduced Ejection Fraction (DEFINE-HF) clinical trial [18]. Even though this trial included only a small sample of patients and patients exclusively from the United States, it has been concluded that the benefits of dapagliflozin in patients with established heart failure and a reduced ejection fraction also apply to patients without diabetes [18].

In a recent six-month experiment, the effects of the SGLT2 inhibitor dapagliflozin on proteinuria in non-diabetic patients with chronic kidney disease (Empagliflozin in Non-diabetic Patients With Heart Failure and Reduced Ejection Fraction (EMPA-TROPISM)) done only on non-diabetics (n=84) with HFrEF revealed that the benefits of EMPA extend beyond diabetes, particularly in terms of CV outcomes. In its primary and secondary end points, this study clearly demonstrated an improvement in patients with heart failure reduced ejection fraction HFrEF by reducing left ventricular (LV) volume, decreasing LV mass, increasing LV systolic function, and improving functional capacity and quality of life (QoL) [27]. Despite the favorable results, the study had a small sample size and the impact on heart failure preserved ejection fraction (HFpEF) was not investigated.

Besides, the Empagliflozin in Heart Failure Patients With Reduced Ejection Fraction (EMPIRE-HF) trial, which included 190 diabetic and non-diabetic patients, found no statistical significance in non-terminal prohormone B-type natriuretic peptide (NT-proBNP), QoL, or daily activity levels after providing EMPA 10 mg for three months [26]. On the contrary, subjects in the DAPA-HF study, who had both diabetes and non-diabetes, saw a 20% drop in NT-proBNP following eight months of dapagliflozin medication. This could be owing to the small number of patients in EMPIRE-HF, or it could be because the patient cohort in EMPIRE-HF was effectively treated with other prescribed therapy. However, patients in DEFINE-HF showed no statistical significance in terms of NT-proBNP alteration after 12 weeks of treatment with DAPA. As a result, it backs up the evidence that DAPA and EMPA had no effect on NT-proBNP. Thus, the contrasting effects of SGLT-2i in DAPA-HF from DEFINE-HF are explained by the mechanism of diuretic effect due to an increase in hematocrit or reduction of afterload due to decreased systolic pressure which together decreases the release of NT-proBNP [37].

Moreover, results from DAPA-HF have proved that SGLT-2i have beneficial effects on morbidity and mortality in patients with HFrEF. Therefore, end points in EMPIRE-HF should have been addressed appropriately. More research on a large cohort of patients with appropriate objectives is needed in the future to have a better understanding. Next, a small RCT trial, the Effects of the SGLT2 Inhibitor Dapagliflozin on Proteinuria in Non-diabetic Patients With Chronic Kidney Disease (DIAMOND trial), on non-diabetes patients with proteinuric kidney disease found no significant benefits [25]. Because there was a fewer sample size and still large trials are underway.

There were two smaller trials in non-diabetic kidney disease patients that were not included due to the small sample size and lack of statistical significance. DAPA on top of renin-angiotensin-aldosterone system (RAAS) blocking medication produced neutral renal hemodynamic and antiproteinuric effects in non-diabetic patients with focal segmental glomerulosclerosis (FSGS), according to the initial pilot trial by Rajasekeran et al., which included 10 non-diabetic patients [38]. The second research, conducted by Bays et al., enrolled 370 non-diabetic obese patients who did not exhibit any renal improvements after 12 weeks of Canagliflozin 100 or 300 mg/day treatment [39].

In the future, the Study of Heart and Kidney Protection With Empagliflozin (EMPA-KIDNEY) will also give further information to healthcare professionals about the use of SGLT-2i in patients with CKD who do not have T2DM, as well as in patients with more advanced CKD (stage 3b and early stage 4) who do not have albuminuria [40]. Additionally, more details from the Empagliflozin Cardiovascular Outcome Event Trial in Type 2 Diabetes Mellitus Patients (EMPEROR-PRESERVED) [41] major outcome trial, which included non-diabetic patients, might shed more light on the SGLT-2i empagliflozin's efficacy outside of diabetes.

Limitations

There are several limitations to this study that should be noted. To begin, we only looked at randomized controlled trials, systematic reviews, and meta-analyses published in the last 10 years. To our knowledge, there are no larger clinical trials designed specifically for non-diabetic individuals. Furthermore, the sample size of non-diabetic cohorts in current studies is smaller. As a result, we included RCTs and studies involving both diabetic and non-diabetic individuals. Also, as a safety profile, we did not address the negative impacts of SGLT-2i. Finally, the RCTs examined in this article varied in terms of study design, patient cohort, and primary and secondary outcomes.

## Conclusions

In terms of cardiovascular and renal outcomes, sodium-glucose cotransporter-2 inhibitors (SGLT-2i) have been shown to be effective. The cardiovascular and renal advantages of SGLT-2i in non-diabetic patients were the focus of this systematic study. Although no large clinical trial has been conducted exclusively on non-diabetics, recent trials reporting cardiovascular (CV) and renal outcomes in a cohort of both diabetes and non-diabetics have shown a significant reduction in heart failure (HF) hospitalization and mortality from CV causes, as well as prevention of chronic kidney disease progression and death. Surprisingly, identical efficacy has been reported in both populations (diabetes and non-diabetes). The results of ongoing and future randomized controlled trials will undoubtedly broaden the clinical indications for SGLT-2i in a wide range of patient populations, allowing us to discover and unlock their utility beyond anti-glycemic effects. However, more studies with a larger non-diabetic sample size are needed in the future. This research could be groundbreaking, opening new information and future research questions on the effects of SGLT-2i on cardioprotective and renoprotective results in non-diabetic people.
